# Outcome of a novel modified endoscopic papillectomy for duodenal major papilla adenoma

**DOI:** 10.1007/s00464-020-07715-0

**Published:** 2020-07-14

**Authors:** Pin Wang, Chengfei Jiang, Yi Wang, Lin Zhou, Shu Zhang, Xiwei Ding, Ying lv, Lei Wang, Xiaoping Zou

**Affiliations:** 1 Department of Gastroenterology, Nanjing Drum Tower Hospital, Affiliated Hospital of Nanjing University Medical School, Nanjing, 210008 Jiangsu China; 2 Department of Gastroenterology, Affiliated Drum Tower Hospital, Medical School of Nanjing University, Nanjing, Jiangsu China

**Keywords:** Adenoma, Duodenal major papilla, Endoscopic resection, Modified endoscopic papillectomy

## Abstract

**Background and aims:**

In recent years, with the development of endoscopic techniques, endoscopic resection is widely used for duodenal papillary adenomas, but conventional endoscopic resection has a high rate of incomplete resection and recurrence. On this basis, we have employed a novel modified endoscopic papillectomy (ESP). In this study, we evaluated the feasibility and advantages of this ESP for the treatment of duodenal major papilla adenoma.

**Methods:**

A total of 56 patients with duodenal major papilla adenoma confirmed by endoscopic ultrasonography, intraluminal ultrasound and gastroscopic biopsy from October 2007 to June 2017 were collected in the Department of Gastroenterology, Nanjing Drum Tower Hospital. The diameter of the adenoma ranged from 1.41 to 2.02 cm. 16 cases were given the conventional method and 40 cases underwent the modified ESP procedure in which a small incision was made by cutting current when anchoring the snare tip on the distal side of the adenoma.

**Results:**

En bloc resection rate was significantly higher in the modified group (100%, 40/40) than that in the conventional group (81.3%, 13/16; *P* = 0.02). However, no significance was seen between the modified group and the conventional group in complete resection rate (92.5%, 37/40 vs 93.8%, 15/16; *P* = 1.00). There was no significant difference in the number and difficulty of postoperative pancreatic and biliary stents placement between the two groups (*P* = 0.20). Total bleeding occurrence was much lower in the modified group (37.5%, 15/40 vs 87.5%, 14/16; *P* = 0.001), and no significant differences were found in other short-term complications and the 3, 6, 12 and 24 months recurrences rate between the conventional and modified ESP groups.

**Conclusions:**

The modified ESP improves the treatment outcome of duodenal major papilla adenoma with higher en bloc resection rate and lowering bleeding rate.

**Electronic supplementary material:**

The online version of this article (10.1007/s00464-020-07715-0) contains supplementary material, which is available to authorized users.

The duodenal papilla adenomas are part of the tumors of the ampulla of Vater, with the most common ones being ampullary adenomas situated at the main duodenal papilla [1]. They may occur sporadically or in the setting of familial adenomatous polyposis (FAP) [2]. Although defined as benign, duodenal major papillary adenomas are considered premalignant neoplasms. Therefore, complete resection is crucial for a curative treatment of this disease [3].

Traditionally, duodenal major papillary adenomas are resected surgically either by transduodenal local resection or pancreatoduodenectomy [4]. However, radical surgery is associated with serious surgical trauma, high morbidity and mortality rates, and multiple complications. And even focal excision is still too invasive for small lesions [5]. Therefore, endoscopic papillectomy (ESP) has increasingly become a therapeutic modality of choice for papillary adenomas with less degree of invasion and more safety.

Up to now, the ESP guidelines have not been established yet, and certain issues related to this technique still remain controversial, such as optimal papillectomy procedure and type of snare to be used [6]. The main concerns about routine ESP are the potential risk of incomplete removal, local recurrence and procedure-related complications [7–9]. To address these problems, we recently worked out a modified ESP approach, which is combined with injection of a small amount of methylthioninum chloride (MTC, also called methylene blue) and epinephrine into the submucosa to reveal the lesion more clearly. More importantly, by making a small incision with cutting current, we anchored the snare tip on the distal side of the adenoma [10]. The snare was then opened in a circular shape and held in place without slippage, which could enable the resection of the lesion en bloc [10]. Later, a similar approach was used to treat sporadic duodenal adenomas by Forte et al. [11].

The main objective of this study was to further evaluate the advantages of the above described modified ESP for the treatment of duodenal major papilla adenomas in more patient samples, as compared with the conventional method.

## Methods

### Patients

From October 2007 to July 2017, a total of 56 patients with duodenal major papillary adenoma were treated with ESP in our hospital. Among them, 16 cases from October 2007 to September 2011 were gone through with the conventional method, and 40 cases between January 2012 and June 2017 received ESP with the modified method. The clinical data of all patients were retrieved for this study. All patients signed informed consent before the procedure.

### Preoperative assessments

Imaging examinations including upper abdomen CT and MRI were performed to show lesions and surrounding lymph nodes. Endoscopic ultrasonography (EUS) was performed to provide more information of the depth of tumor invasion, metastasis of lymph nodes and other organ involvement in duodenal major papillary adenomas. Infiltration of the tumor in biliary and pancreatic duct was evaluated by intraductal ultrasonography (IDUS). At least six pieces of tissue were taken for preoperative biopsy. ESP candidates included adenoma or carcinoma in situ confirmed by endoscopic biopsy and endoscopic ultrasonography without invasion of the muscular layer of the wall, bile duct, or pancreatic duct, and lymph node metastasis. For those cases that involved invasion of bile and pancreatic duct, and adjacent lymph node involvement, surgical treatment is required.

### Equipment

The duodenoscope (JF-260 V), EUS (SSD-α5), IDUS (UM-DG20-31R), and snare (SD-210U-25) used for the resection were all from Olympus Optical Company (Tokyo, Japan). High-frequency generator (VIO 200D) was from ERBE USA (Marietta, Georgia).

### ESP methods

First, with indigo carmine or methylene blue staining, the extent and edge of the lesion were evaluated, or by narrow-band imaging (NBI), especially when a diverticulum is anatomically related to the duodenal papilla. Before endoscopic papillectomy, patients were sedated with diazepam (10 mg) and scopolamine (20 mg) intramuscularly or under anesthesia. A methylene blue-saline mixture was injected into the submucosa of the base of the papilla tumor in order to lift the base and separate the mucosal and submucosal layer from the muscular layer.

**Fig. 1:**
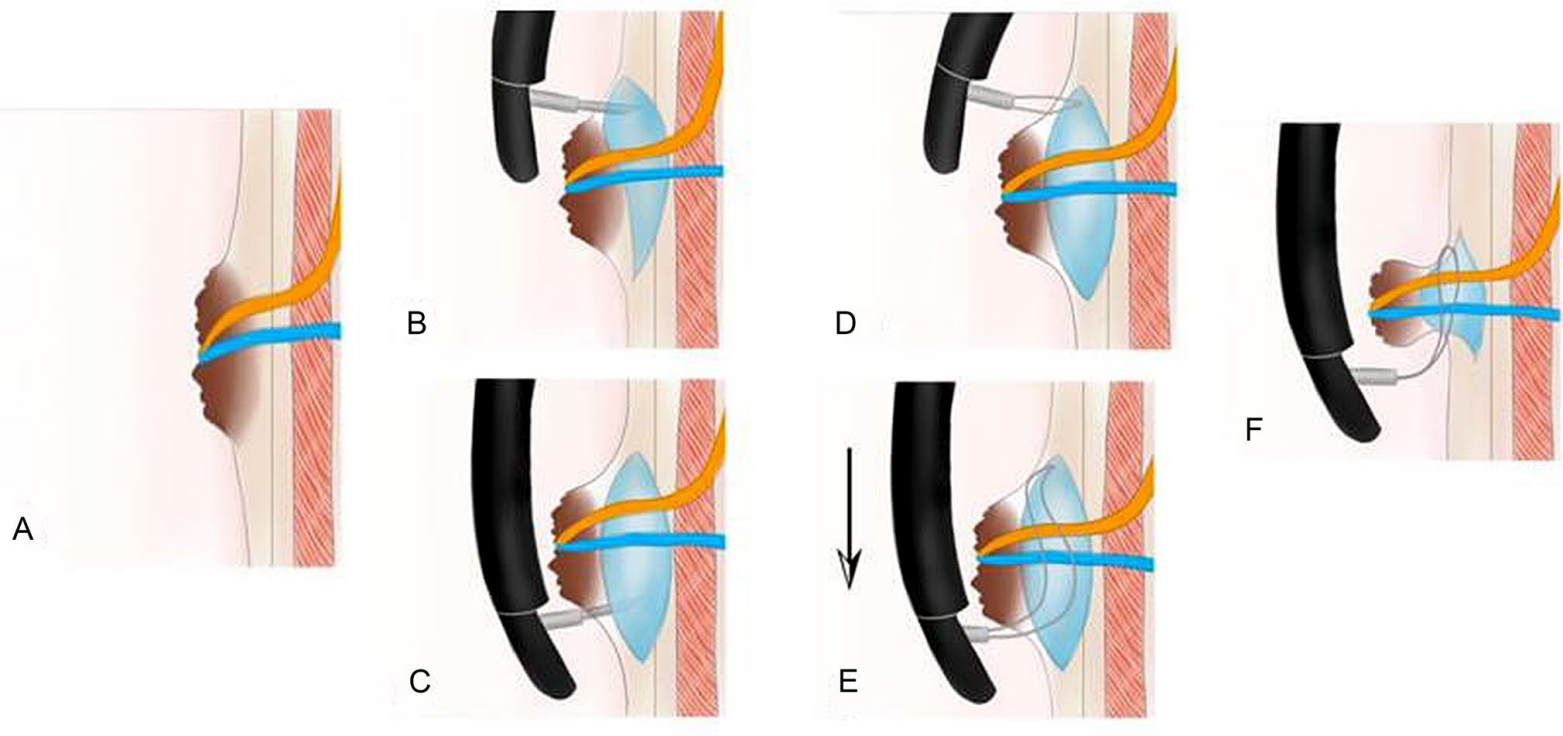
Graphical abstract of modified ESP. **A** Endoscopic manifestation of duodenal major papilla adenoma; **B, C** submucosal injection; **D** small incision was made when anchoring the snare tip against a site which is 0.5 cm away from the upper edge; **E**, **F** Adenoma was trapped using the snare

**Fig. 2:**
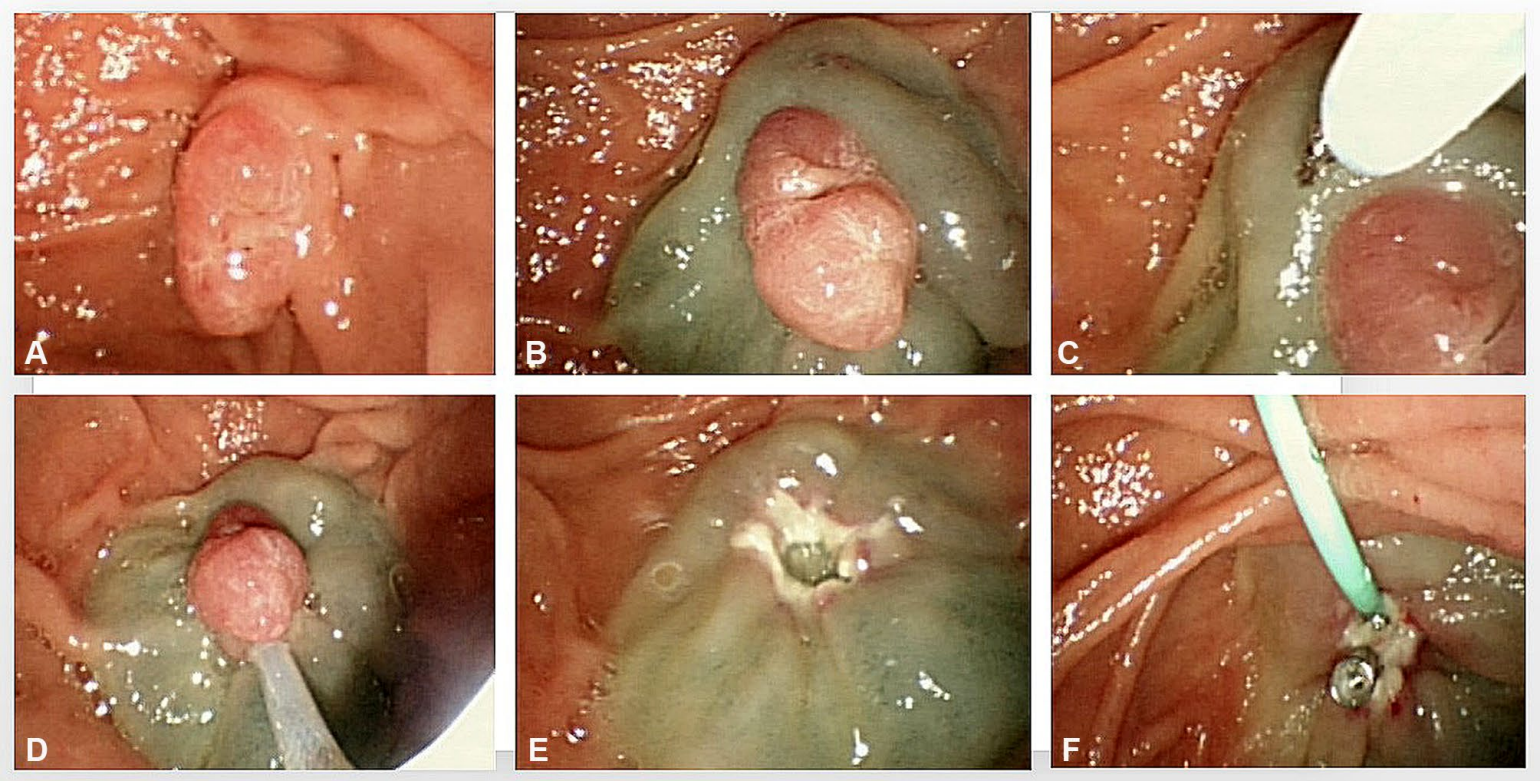
Modified endoscopic papillectomy. **A** Endoscopic manifestation of duodenal major papilla adenoma; **B** submucosal injection; **C** small incision was made when anchoring the snare tip against a site which is 0.5 cm away from the upper edge; **D, E** adenoma was trapped using the snare; **F** pancreatic stent placement

1. Conventional ESP: using the tip of the snare as a fulcrum, we anchored it on one side of the adenoma. The snare was then gradually spread around the lesion and holding it firmly and completely. High-frequency blended electric cutting current (cutting mode: ENDOCUT, effect 2, maximum 90 W) was applied to resect the lesion when the snare was closed maximally.
2. Modified ESP: the basic papillectomy procedure described in our previous report [10] was modified based on the above conventional method. The key difference is that when we anchored the snare tip against a site which is 0.5 cm away from the upper edge of the adenoma, a small incision was made by blended cutting current (cutting mode: ENDOCUT, effect 2, maximum 90 W) and the tip of the snare was gently inserted into the incision. The snare was then slowly released in a circular shape and held in place without slippage. After the lesion was completely trapped, we tightened the snare and finally resected the lesion en bloc (Figs. 1, 2, Supplemental video file).

Endoscopic retrograde cholangiopancreatography (ERCP) and a prophylactic pancreatic and biliary stent placement were attempted after ESP or modified ESP. Bleeding requiring hemostasis was controlled by epinephrine (1:10,000) injection, argon plasma coagulation (APC) or endoscopic hemoclip. All patients were subjected to a short-term fast and monitoring of serum amylase in the postoperative period. In addition, all of them stayed in hospital for 3 to 5 days for observation, during which period complications including hemorrhage and pancreatitis/cholangitis were monitored. The specimens were fixed with 4% formalin and evaluated with histopathology. En bloc resection was defined as the entire lesion being resected in one piece. Complete resection was defined as negative lateral or vertical margins.

### Follow-up

Follow-up endoscopy with biopsies was performed at 3, 6, 12 and 24 months after the procedure, and yearly afterwards. Over the follow-up period, if a new lesion was found to be 1 cm within the original focus and surroundings, the lesion was regarded as residual. If it occurred after 6 months, it would be considered as local recurrence. Sporadic cases were followed up for a minimum of 2 years, and follow-up of patients with FAP took 3 to 5 years.

### Statistical analysis

IBM SPSS Statistics version 22.0 (IBM, Armonk, NY) was utilized to conduct analyses involving Chi-square test and *t* test. Numerical variables were expressed as means ± standard deviation or median. Two-sided *P* values < 0.05 were considered to indicate statistical significance.

## Results

### Baseline characteristics

In this study, a total of 56 patients were enrolled, of which 16 were in the conventional group (male 12, female 4) and 40 in the modified group (male 26, female 14) (Table 1). The mean age was 63.81 in the conventional group and 58.33 in the modified group. The mean age of all patients was 59.89 ± 11.89 years. The overall mean size of tumor was 1.81 ± 0.83 cm in length and 1.45 ± 0.62 cm in width. The length (1.76 ± 0.89 cm vs 2.02 ± 0.57 cm; *P* = 0.10) and width (1.41 ± 0.65 mm vs 1.59 ± 0.50 cm; *P* = 0.24) of the tumor were slightly greater in the conventional group than the modified group, albeit no statistical significance. Of the 56 patients, 16 were adenomas in the conventional group (9 low-grade, 3 moderate-grade, 4 high-grade), and 40 were adenomas in the modified group (27 low-grade, 7 moderate-grade, and 6 high-grade). The median number of white blood cells in the conventional group was 5.71 ± 1.72 ml, and the mean hemoglobin concentration was 130 g/l. The median number of white blood cells in the modified group was 5.81 ± 1.66 ml, and the average hemoglobin concentration was 128 g/l. The conventional and modified groups are comparable in that the patient gender, age, tumor size, biopsy result, white blood cell count and hemoglobin all exhibited no significant statistical difference (*P* > 0.05).

**Table 1:** Baseline characteristics

Variable	Conventional group (*n* = 16)	Modified group (*n* = 40)	*P* value
Sex of male (%)	12 (75.0)	26 (65.0)	0.54
Age (year)	63.81 ± 9.43	58.33 ± 12.50	0.12
Size (cm)			
Length	2.02 ± 0.57	1.76 ± 0.89	0.10
Width	1.59 ± 0.50	1.41 ± 0.65	0.24
Biopsy result			0.58
Low-grade	9 (56.3)	27 (67.5)	
Moderate-grade	3 (18.8)	7 (17.5)	
High-grade	4 (25.0)	6 (15.0)	
White blood cell count (/ml)	5.71 ± 1.72	5.81 ± 1.66	0.89
Hemoglobin (g/l)	130 ± 25.68	128 ± 24.65	0.81

### Comparison of outcomes of two papillectomy methods

Of the 16 cases in the conventional group, complete resection was achieved in 15 patients with negative lateral and vertical margins in the pathological examination (Table 2). Dysplasia was observed in the lateral resection margins of 1 patient and this patient was not taken into account. In the modified group containing 40 cases, complete resection was achieved in 37 patients, while three failed and underwent APC (Table 2). The rate of complete resection of adenoma in the conventional group (93.8%, 15/16) and modified group (92.5%, 37/40) was not significantly different (*P* = 1.00). However, in the conventional group, the adenomas were resected en bloc in 13 (81.3%) patients and piecemeal in 3 (18.8%). En bloc resection rate in the modified group was 40 out of 40 patients (100%), which was significantly higher than that of the conventional group (*x*^2^ = 7.925, *P* = 0.020).

**Table 2:** Papilectomy outcome

	Total (*n* = 56)	Conventional group (*n* = 16)	Modified group (*n* = 40)	*P* value
Resection method				0.02
En bloc	53	13	40	
Piecemeal	3	3	0	
Stent insertion				0.14
Without duct	3 (5.9)	1 (7.7)	2 (5.3)	
Common bile duct	1 (2.0)	0 (0.0)	1 (2.6)	
Pancreatic duct	35 (58.6)	6 (46.2)	29 (76.3)	
Both pancreatic bile duct	12 (23.5)	6 (46.2)	6 (15.8)	
Final diagnosis				0.30
Adenoma low-grade	43 (76.8)	11 (68.8)	32 (80.0)	
Adenoma high-grade	8 (14.3)	2 (12.5)	6 (15.0)	
Adenocarcinoma	5 (8.9)	3 (5)	2 (5.0)	
Complete resection	52 (92.9)	15 (93.8)	37 (92.5)	1.00

### Effects of stent insertion

After tumor removing, the biliary and pancreatic stents were placed depending on the situation to prevent possible complications. However, the resection of lesion could add some difficulty to stent placement. Of 13 patients undergoing conventional resection (excluding 3 with carcinoma from 16 patients), both biliary and pancreatic stents were placed in 6, pancreatic stent alone in 6 and no stent placement in 1 case. Of the 38 patients in the modified group, both biliary and pancreatic stents were placed in 6, biliary stent alone in 1, pancreatic stent alone in 29, and no stent placement in 2 cases. The results showed that the number and difficulty of stent placement between two groups had no statistic difference (Table 2).

### Short-term complications related to ESPs

We compared the incidence of short-term complications (3 months after papillectomy) between the methods in Table 3. Post-papillectomy bleeding was observed in 29 (51.8%) patients totally: 14 (87.5%) in the conventional group and 15 (37.5%) in the modified group, showing a significant reduction when the modified ESP was employed (*P* = 0.001). In general, the overall rate of delayed bleeding was much lower compared with that of immediate bleeding (35.7% vs. 7.1%). Although no patient with the conventional ESP suffered from delayed bleeding only, the total delayed bleeding was 25% in the conventional group and 12.5% in the modified group when patients with delayed bleeding only and with both types of bleeding were calculated together (Table 3). Bleeding was mostly controlled by APC (13.8%, 4/29) or hemoclip application (31.0%, 9/29). Perforation was seen in 1 case in the conventional group and 2 cases in the modified group. Post-papillectomy pancreatitis occurred in 4 (7.1%) patients totally: 1 (6.3%) in the conventional group and 3 (7.5%) in the modified group (*P* = 1.00). Post-papillectomy cholangitis occurred in 5 (8.9%) patients totally: 1 (6.3%) in the conventional group and 4 (10.0%) in the modified group (*P* = 1.00). All the complications were relieved after conservative medical therapy.

**Table 3:** Papillectomy-related adverse events

	Total (*n* = 56)	Conventional group (*n* = 16)	Modified group (*n* = 40)	*P* value
Bleeding	29 (51.8)	14 (87.5)	15 (37.5)	0.001
Bleeding type				
Immediate bleeding	20 (35.7)	10 (62.5)	10 (25.0)	
Delayed bleeding	4 (7.1)	0 (0)	4 (10.0)	
Both	5 (8.9)	4 (25.0)	1 (2.5)	
Perforation	3 (5.4)	1 (6.3)	2 (5.0)	1.00
Pancreatitis	4 (7.1)	1 (6.3)	3 (7.5)	1.00
Cholangitis	5 (8.9)	1 (6.3)	4 (10.0)	1.00

When analyzed for pancreatic stent insertion status, pancreatitis was in 7.8% (4/51) of patients with successful pancreatic stent insertion and no patients had pancreatitis when unsuccessful (Table 4). In addition, no significant difference was seen with regard to cholangitis (*P* = 0.60).

**Table 4:** Occurrence of pancreatitis/cholangitis according to pancreatic/biliary stent insertion status

	Total (*n* = 56)	Successful pancreatic stent insertion (*n* = 51)	Unsuccessful pancreatic stent insertion (*n* = 5)	*P* value
Pancreatitis	4 (7.1)	4 (7.8)	0 (0)	1.00

	Total (*n* = 56)	Successful biliary stent insertion (*n* = 15)	Unsuccessful biliary stent insertion (*n* = 41)	*P* value
Cholangitis	5 (8.9)	2 (13.3)	3 (7.3)	0.60

### Recurrence and residual lesion after ESP

Among all the patients selected initially, we were unable to carry out follow-up studies for 8 of them as they refused to accept surveillance endoscopy (4 in conventional group and 4 in modified group), and 5 patients with subsequent surgery for the pathological diagnosis of duodenal papillary adenocarcinoma were also excluded. Therefore, 43 patients were included for follow-up, with adenomas of low-grade, moderate-grade, or high-grade dysplasia. In the conventional group, no postoperative residual lesion was found at 3-month of follow-up, 1 case found at 6 months and 1 case found at 24 months (Table 5). With respect to 34 patients in the modified group, 2 case showed residual lesion at 3 months, 0 case showed recurrence at both 6 months and 12 months, 3 case showed recurrence at 24 months. At the 24-month follow-up, the recurrence rates were 22.2% (2/9) and14.7% (5/34) in the conventional and modified groups, respectively, and the difference was not statistically significant (*P* = 0.624).

**Table 5:** Tumor persistence after papillectomy

	Total (*n* = 43)	Conventional group (*n* = 9)	Modified group (*n* = 34)	*P* value
Positive biopsy at 3 months	2 (4.6)	0 (0.0)	2 (5.9)	1.00
Positive biopsy at 6 months	1 (2.3)	1 (11.1)	0 (0.0)	0.21
Positive biopsy at 12 months	0 (0.0)	0 (0.0)	0 (0.0)	
Positive biopsy at 24 months	3 (7.0)	1 (11.1)	3 (8.8)	1.00

## Discussion

The detection rate of ampullary neoplasms has been increased by the widespread use of esophago-gastro-duodenoscopy (EGD), ultrasonography (US) and ERCP. Given its potential malignant transformation, complete en bloc resection of papilla adenoma should be given as soon once it is found. Endoscopic resection of the duodenal major papilla adenomas is a safe and efficacious first-line therapy [4]. It was first described by Suzuki et al. [12] and the first large sample study was described by Binmoeller et al. [13, 14]. Because of its low morbidity and mortality [15–21], it has become an alternative to surgical therapy. However, its recurrence and residual rates are still high, with a recurrence of 24% according to Binmoeller et al. [13] and 33% by Cheng et al. [22]. The most important reason could be the difficulty to resect en bloc in the duodenum. In the past, it was generally considered that lesions within 2 cm could be completely trapped by a snare and resected in an en bloc manner while lesion larger than 2 cm may have to be removed by piecemeal resection. The procedure is usually challenging, as the duodenal space is small with several adjacent organs. In addition, the snare does not have a real fulcrum to hold itself in place without slippage to completely trap the lesions, especially for larger papillary adenomas, which could lead to increased chances of residual lesions and recurrence rates.

To tackle the above described issue, we developed a modified duodenoscopic papillomaectomy which we also called duodenal papilla adenoma fenestration to increase en bloc resection rate and to reduce recurrence and residual risk [10]. Compared to the report of two cases with sporadic duodenal adenomas in that anchoring the snare tip with a small incision in the submucosa facilitates en bloc endoscopic mucosal [11], our approach has the following differences: we focused on the duodenal major papilla adenoma with an increased sample size and employed a duodenoscopy instead of an esophago-gastro-duodenoscopy to carry out the procedure. The use of duodenoscopy has several advantages: (1) Side-view endoscope makes it easier to get a glimpse of the entire appearance of the papilla and adenomas, especially to identify the location of the papilla openings; (2) Skilled endoscopists can adjust the injection needle, snare and titanium by means of the forcep elevators and angulation knob to complete the procedure under direct-vision; (3) After the adenoma is removed, the direct insertion of the biliary and pancreatic ducts can be achieved without the replacement of the endoscope. Local scars after adenoma resection leading to obstruction of biliary and pancreatic ducts can be avoided.

This work demonstrated that using either a modified or conventional ESP is effective with regard to the rate of complete resection. The total rate was 92.9%, which is on the top end of those described in previous reports (46–92%) [23]. When it comes to specific aspects, the complete resection rate did not differ much (93.8% in the conventional group vs 92.5% in the modified group), indicating that the modified method had no significant effect on complete resection rate. In contrast, the modified ESP dramatically improved the en bloc resection rate (100%) as compared with the conventional group (81.3%). Such a result meets our expectation that making a small incision before resection would open the snare in a circular shape and taut around the lesion without slippage, making it easier to resect the lesion en bloc.

Interestingly, at 3 months of procedure the residual rate showed no difference between the two groups (0% vs 5.9%, conventional vs. modified), despite of the initial difference in the en bloc resection rate. One reason could be the efforts spared in resect attempts, no matter it was en bloc or piecemeal. On top of that, thermal ablation including APC may also decrease rate of lesion residue. As acknowledged by Catalano et al. [24], thermal ablation is an effective auxiliary method for the success of papillectomy. This might explain the low residue rate in the conventional group even though there is lower en bloc resection rate.

There have been disputes over the application of submucosal injection during endoscopic resection of papilloma of the duodenum. Such injection is quite beneficial for tumors originated from the papilla and growing to the duodenal mucosa (> 1 cm) or for lesions located at the edge or within a diverticulum, since these features may facilitate the use of snare owing to an easy lifting of the lesion and clear visual field [4]. As with larger-sized lesions, the injection usually begins from its distal end, then the lateral areas and the proximal end. For those lesions located in the edge or within a diverticulum, submucosal injection should be initiated from the edge of the lesion in the diverticulum. It should be noted that the lesion lifting using submucosal injection may not be achieved as there are bile ducts and pancreatic ducts passing through the papilla area. Furthermore, a complete lifting of surrounding normal mucosa may occur after injection, leading to embedding of the central area of the lesion in the surrounding normal mucosa. Consequently, the snare cannot be placed well, impeding the successful performance of mucosal resection. Nevertheless, submucosal injection can reduce the incidence of perforation and postoperative complications resulted from endoscopic resection. It can also assist to judge the depth of infiltration. Therefore, we support the application of submucosal injection before resection. It is worth mentioning that appropriate fluid volume in submucosal injection should be given, as due to a narrow luminal cavity of the duodenum, an over-raised mucosa may affect the visual field observation and the use of snare.

Whether or not the pancreaticobiliary stent needs to be placed after the removal of the duodenal adenoma is an issue to be explored. The thermal damage caused by endoscopic resection leads to pancreatic duct edema, and the poor pancreatic drainage is one of the important causes of pancreatitis. Therefore, some investigators believe that pancreatic duct sphincter incision should be performed immediately after endoscopic papillectomy. This could reduce the incidence of pancreatitis as well as the risk of fibrosis and stenosis in the pancreatic duct opening. Some even propose that all patients should be placed in the pancreatic duct stent. In this study, most of the cases were placed on the pancreatic duct stent.

As for the short-term complications, we found that the occurrence of bleeding was significantly different between the two groups. The frequency of bleeding was significantly higher in the conventional group (87.5%) than that in the previous studies (37.5%) [23]. The reason might be that we included immediate bleeding in this work but no information about such inclusion is available in other studies. Given that the delayed bleeding is more important than immediate bleeding in clinical significance, our results demonstrated that the incidence of total delayed bleeding in the modified group (12.5%) was only half of that of the conventional group (25%), when combining the patients with delayed bleeding only with those having both types of bleeding, To reach a conclusion, larger-sized research is needed to validate the relevance in bleeding events.

As for other complications including perforation, pancreatitis and cholangitis, no significant difference was observed between the two methods. The analysis based on the stent insertion showed similar results too (*P* > 0.05). However, this finding conflicted with previous reports recommending stent placement to reducing related complications [13, 20]. Thus, studies with larger patient numbers are needed to address this discrepancy.

In conclusion, this study showed that endoscopic resection of duodenal major papillary adenoma with anchoring the snare tip using a small incision is beneficial to resect duodenal major papilla adenoma en bloc. This method would also reduce the frequency of bleeding. In addition, no significant differences were seen in other post-procedure adverse events, the complete resection rate or recurrence rate between the modified and conventional method. A prospective, randomized multicenter and large-size sample study is warranted to validate our findings reported in the future.

## Electronic supplementary material

Below is the link to the electronic supplementary material.

Supplementary file1 (MP4 15154 kb)
